# Increased Expression of Plasma-Induced *ABCC1* mRNA in Cystic Fibrosis

**DOI:** 10.3390/ijms18081752

**Published:** 2017-08-11

**Authors:** Justin E. Ideozu, Xi Zhang, Amy Pan, Zainub Ashrafi, Katherine J. Woods, Martin J. Hessner, Pippa Simpson, Hara Levy

**Affiliations:** 1 Division of Pulmonary Medicine, Department of Pediatrics, Ann & Robert H. Lurie Children’s Hospital of Chicago, 225 E Chicago Ave., Chicago, IL 60611, USA; jideozu@luriechildrens.org (J.E.I.); XiZhang@luriechildrens.org (X.Z.); ZAshrafi@luriechildrens.org (Z.A.); 2 Human Molecular Genetics Program, Stanley Manne Children’s Research Institute, Chicago, IL 60614, USA; 3 Department of Pediatrics, Division of Quantitative Health Sciences, Medical College of Wisconsin, Milwaukee, WI 53226, USA; apan@mcw.edu (A.P.); psimpson@mcw.edu (P.S.); 4 Department of Pediatrics, Division of Critical Care, Medical College of Wisconsin, Milwaukee, WI 53226, USA; kawoods@mcw.edu; 5 Department of Pediatrics, Max McGee National Research Center for Juvenile Diabetes, Medical College of Wisconsin, Milwaukee, WI 53226, USA; mhessner@mcw.edu; 6 Northwestern University Feinberg School of Medicine, Chicago, IL 60611, USA

**Keywords:** *ABCC1*, cystic fibrosis, DNA methylation, polymorphism

## Abstract

The *ABCC1* gene is structurally and functionally related to the cystic fibrosis transmembrane conductance regulator gene (*CFTR*). Upregulation of *ABCC1* is thought to improve lung function in patients with cystic fibrosis (CF); the mechanism underlying this effect is unknown. We analyzed the *ABCC1* promoter single nucleotide polymorphism (SNP rs504348), plasma-induced *ABCC1* mRNA expression levels, and *ABCC1* methylation status and their correlation with clinical variables among CF subjects with differing *CFTR* mutations. We assigned 93 CF subjects into disease severity groups and genotyped SNP rs504348. For 23 CF subjects and 7 healthy controls, donor peripheral blood mononuclear cells (PBMCs) stimulated with plasma underwent gene expression analysis via qRT-PCR. *ABCC1* promoter methylation was analyzed in the same 23 CF subjects. No significant correlation was observed between rs504348 genotypes and CF disease severity, but pancreatic insufficient CF subjects showed increased colonization with any form of *Pseudomonas aeruginosa* (OR = 3.125, 95% CI: 1.192–8.190) and mucoid *P. aeruginosa* (OR = 5.075, 95% CI: 1.307–28.620) compared to the pancreatic sufficient group. A significantly higher expression of *ABCC1* mRNA was induced by CF plasma compared to healthy control plasma (*p* < 0.001). CF subjects with rs504348 (CC/CG) also had higher mRNA expression compared to those with the ancestral GG genotype (*p* < 0.005). *ABCC1* promoter was completely unmethylated; therefore, we did not detect any association between methylation and CF disease severity. In silico predictions suggested that histone modifications are crucial for regulating *ABCC1* expression in PBMCs. Our results suggest that *ABCC1* expression has a role in *CFTR* activity thereby increasing our understanding of the molecular underpinnings of the clinical heterogeneity in CF.

## 1. Introduction

Cystic fibrosis (CF) is a heritable multisystem disorder caused by mutations in the cystic fibrosis transmembrane conductance regulator (*CFTR*) gene [1,2]. Despite advances in newborn screening and treatments for CF, clinical heterogeneity remains a major challenge [3]. Modifier genes that may impact CF disease severity are emerging keys to deciphering clinical heterogeneity [4,5,6,7].

The gene encoding the multidrug resistance-associated protein 1, *ABCC1/MRP1*, is a candidate for further molecular investigation based on its structural and functional association with *CFTR* [8,9]. *ABCC1*, as well as *CFTR* (*ABCC7*) and 11 other genes associated with multidrug resistance, are subfamily C members of the ATP-binding cassette (ABC) transporter genes [10]. The diverse activities of subfamily C transporters include transportation of chemotherapeutic agents, amino acids, glutathione conjugates, and small peptides, as well as excretion of fungal and bacteria toxins [10,11,12]. *CFTR* is unique among the ABC subfamily C members of transporters due to its intrinsic ability to conduct chloride ions at a fast rate [12,13,14], but it shares its closest homology with *ABCC1* [9,15,16].

Since the cloning of *ABCC1* in the early 90s [17,18,19], progress has been made towards establishing a functional relationship between *ABCC1* and *CFTR*. For example, functional complementation of dysfunctional *CFTR* by *ABCC1* following chemotherapy that resulted in increased expression of *ABCC1* was associated with improved lung function in a CF patient [15]. A study analyzing nasal epithelial cells in CF patients also showed that low *ABCC1* transcript levels were associated with more severe disease [8]. Additionally, increased expression of *ABCC1* in nasal cells of CF patients following treatment with azithromycin was associated with restoration of chloride channel conduction and improved lung function [20].

Polymorphisms or epigenetic modifications that alter the expression *ABCC1* may underlie the clinical heterogeneity in lung function observed among CF patients. A functional single nucleotide polymorphism (SNP) in the promoter region of *ABCC1* (rs504348) has been shown to modulate gene expression and impact lung function [21,22]. Mafficini, et al. [23] recently reported that this polymorphism is associated with disease severity in CF patients homozygous for the F508del mutation. However, this study has not been replicated using a cohort of CF patients with differing genotypes, and it is unclear whether this SNP impacts lung function by modulating *ABCC1* expression.

DNA methylation is a predominant epigenetic mechanism that modulates gene expression [24]. Methylation of cytosine-phosphate-guanosine (CpG) dinucleotides present in CpG islands within gene promoters can inactivate and silence gene expression [25,26,27]. The *ABCC1* promoter contains CpG islands that are potential targets for methylation analysis. Previous research in cancer cells has shown that the *ABCC1* promoter is hypomethylated but that the methylation status is not correlated with *ABCC1* gene expression [28]. Whether this epigenetic mechanism modulates *ABCC1* expression in CF is unknown.

Analysis of peripheral blood cell gene expression signatures provides us with a non-invasive approach to diagnose many diseases, including CF. This may involve direct profiling of transcripts of peripheral blood mononuclear cells (PBMCs) from affected individuals [29,30]. Alternatively, analysis of gene expression changes after exposure of healthy donor PBMCs to patient serum or plasma has been used as a model in diabetes, Crohn’s disease, ulcerative colitis and juvenile rheumatoid arthritis [31,32,33,34]. To examine the association between *ABCC1* expression and lung function in CF, we analyzed the functional *ABCC1* promoter polymorphism (rs504348), plasma-induced *ABCC1* expression in PBMCs, and *ABCC1* promoter methylation status among CF subjects with differing *CFTR* genotypes and then examined the correlation of these molecular measures with clinical status.

## 2. Results

### 2.1. Baseline Characteristics of Subjects

A total of 93 CF subjects were recruited for this study. The median (interquartile range (IQR)) subject age was 10 (6, 19) years and 47.3% were males. The majority of CF subjects (74.2%) were pancreatic insufficient. More than half of the CF subjects (58.1%) were positive for *Pseudomonas aeruginosa*, of which 59.2% were positive for the mucoid form of *P. aeruginosa*. The median (IQR) sweat chloride level was 103 (87, 114) mmol/L, and the forced expiratory volume in 1 second (FEV1) percent predicted was 97 (72, 111). Spirometry information and culture results were recorded during the same clinical visit and the collection date was used to deduce sample age. Based on the adopted Epidemiologic Study of Cystic Fibrosis (ESCF) disease severity equation, most CF subjects (47.3%) had normal/very mild disease, while 19 CF subjects (20.4%) had severe disease. Fourteen percent of subjects were not assigned a severity group because they had no recorded FEV1 to estimate FEV1 percent predicted (Table 1).

### 2.2. Association between Disease Severity Categories and Clinical Status

Pancreatic insufficient CF subjects had an increased risk of colonization with any form of *P. aeruginosa* (OR = 3.125, 95% CI: 1.192–8.190, *p* < 0.02) and colonization with mucoid *P. aeruginosa* (OR = 5.075, 95% CI: 1.307–28.620, *p* < 0.012) compared to the pancreatic sufficient group. Association between disease severity (defined by ESCF categories: Moderate and Severe; Mild and Normal/very mild) and pancreatic status, colonization with *P. aeruginosa*, or with mucoid *P. aeruginosa* was assessed using a Chi-Square or Fisher’s exact test. All 3 groups were associated with disease severity (*p* < 0.01). Pancreatic sufficient CF subjects were more likely to have Mild or Normal/mild disease severity than the pancreatic insufficient group (*p* < 0.01). CF subjects in the normal/very mild disease severity group were less likely to have mucoid *P. aeruginosa* colonization compared to CF subjects in the Moderate or Severe ESCF categories (*p* < 0.002) (Table 2).

### 2.3. Frequency Distribution of ABCC1 SNP rs504348 and Association with Clinical Status

Among the 93 CF probands, the observed frequencies for the rs504348 CC, CG, and GG genotypes were 8.6%, 25.8%, and 65.6%, respectively. The distribution was similar (*p* > 0.25) to those reported for the global population and Utah residents (rs504348 genotype distribution from the 1000 genome project can be retrieved from the Ensemble genomic browser at http://www.ensembl.org). Significant deviation from Hardy–Weinberg equilibrium (HWE) was observed in the global population (*p* < 0.0001), Utah residents (*p* < 0.03), and our study cohort (*p* < 0.03). The GG genotype was the most prevalent regardless of ESCF severity classification, pancreatic status, and colonization with *P. aeruginosa*; however, there was no association between any of the subject groups and rs504348 (C/G) genotypes (*p* > 0.05) (Table 3).

### 2.4. Analysis of Plasma-Induced ABCC1 mRNA Expression in CF

Induced *ABCC1* mRNA expression in PBMC exposed to plasma from 23 CF subjects and 7 healthy controls were measured by RT-qPCR. The median and IQR age for CF subjects and healthy controls were 9.4 (6.7, 17.3) and 26.9 (7.0, 27.1) years, respectively. Just over half (56.5%) of the CF subjects were pancreatic insufficient. The *ABCC1* SNP rs504348 was recorded in 78.3% of the CF subjects while 21.7% harbored the ancestral GG genotype (Table 4).

**Figure 1:**
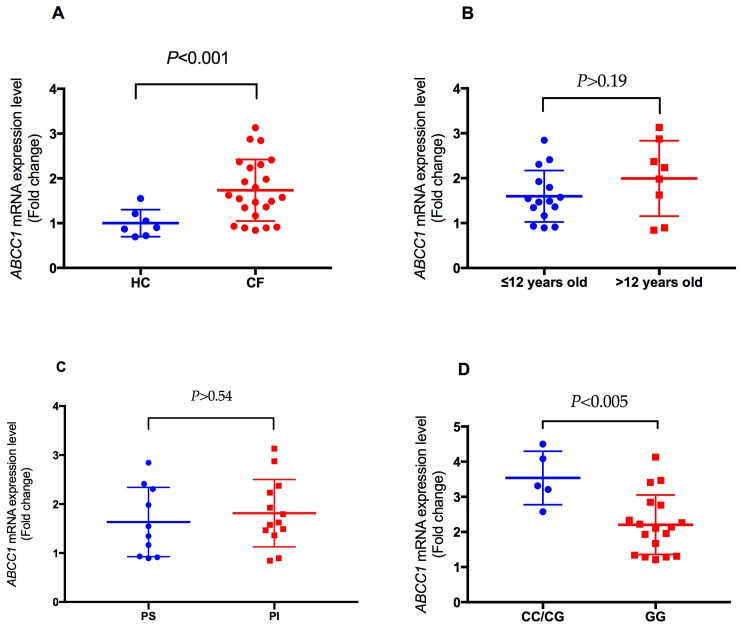
Comparison of plasma-induced *ABCC1* mRNA expression levels in PBMC. (**A**) Expression levels were higher for cystic fibrosis (CF) subjects than healthy controls (HC). No difference was observed between young (≤12 years of age) and old (>12 years of age) CF subjects (**B**) or between pancreatic insufficient (PI) and pancreatic sufficient (PS) CF patients (**C**). Significantly higher *ABCC1* expression was induced with plasma from CF subjects with rs504348 (CC/CG) compared to homozygotes with the ancestral G allele (**D**). Significance estimated using *t*-tests.

Plasma from CF subjects induced a significantly higher *ABCC1* mRNA expression level compared to plasma from healthy controls (*p* < 0.001) (Figure 1A). To rule out any age effect on plasma-induced expression levels by CF subjects, we compared the expression levels between young (≤12 years of age) and old (>12 years of age) CF subjects. We found no significant difference in plasma-induced *ABCC1* expression between the two groups (*p* > 0.19) (Figure 1B). Although plasma-induced expression was higher for pancreatic insufficient CF subjects than pancreatic sufficient CF subjects, the difference in expression levels was not significant (*p* > 0.54) (Figure 1C). However, plasma from CF subjects with rs504348 (CC/CG) induced significantly higher *ABCC1* expression compared to plasma from subjects with the ancestral GG genotype (*p* < 0.005) (Figure 1D), which suggests a *cis*-expression quantitative trait loci (*cis*-eQTL) effect of rs504348 (Figure 1).

### 2.5. ABCC1 Promoter Methylation

**Figure 2:**
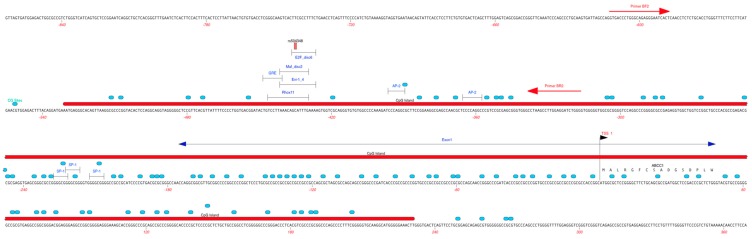
Annotation of the *ABCC1* promoter region targeted for methylation analysis. Red line, CpG island spanning −531 to +231 bp. BF2 and BR2 primers targeting CpG sites (blue circles) and regulatory factors (delimited lines), including the rs504348 (−435 bp) SNP were used for bisulfite PCR.

**Figure 3:**
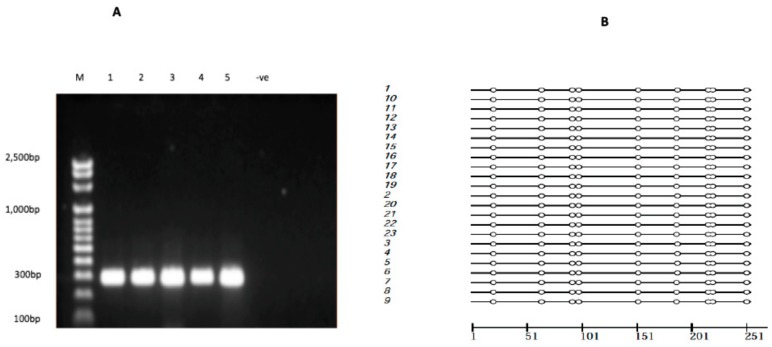
ABCC1 promoter methylation analysis. (**A**) *ABCC1* promoter bisulfite PCR products. Clear bands of ~295 bp shown in lanes 1–5 indicate successful bisulfite PCR. −ve represents unmodified human genomic DNA used as a negative control. (**B**) White cycles reported by Methtools represent unmethylated CpG sites [35].

A 295-bp promoter region of *ABCC1* (NG_028268.1) spanning −612 to −317 bp, containing a predicted CpG island (Figure 2), was amplified from 23 CF subjects using bisulfite PCR (Figure 3A) to determine the methylation status in peripheral blood tissue. Analysis of bisulfite sequence data indicated that the *ABCC1* promoter was completely unmethylated in all CF subject samples analyzed (Figure 3B), indicating that promoter methylation may not be involved in regulating *ABCC1* expression in peripheral blood of CF patients.

### 2.6. Regulatory Effects of rs504348

**Figure 4:**
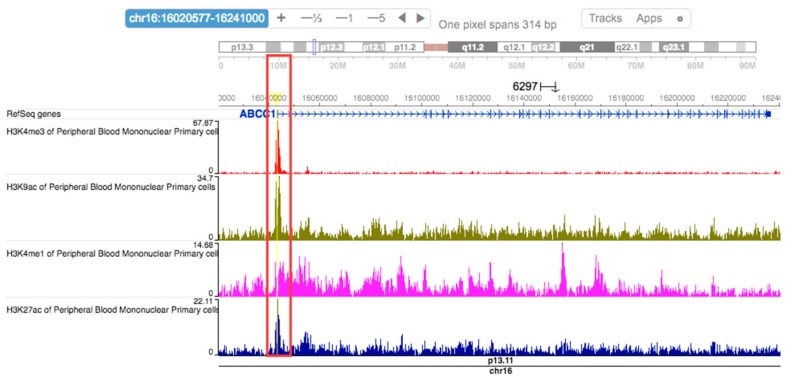
WashU Epigenome Browser schematic of histone marks enriched at the *ABCC1* promoter SNP rs504348. Peaks at H3K4me3, H3K9ac, H3K4me1, and H3K27ac (highlighted in red box) indicate that these histone modifications contribute to the chromatin state assignment at rs504348 in PBMCs and validated results from computational predictions using HaploReg v4.1.

Computational predictions using HaploReg v4.1 [36] showed that the rs504348 SNP overlaps regulatory features of the *ABCC1* promoter, including promoter histone marks, enhancer histone marks, DNAse 1 hypersensitive sites, transcription factor binding sites, and altered regulatory motifs in many tissues (Table 5). In PBMCs, a high cluster of *ABCC1* promoter activity in the region spanning rs504348 was reported by HaploReg v4.1. Histone marks associated with active promoters (H3K4me3 and H3K9ac) and active enhancers (H3K4me1 and H3K27ac) were enriched in the region spanning rs504348, but no enrichment of repressive histone marks was observed in this region (Figure 4). Analysis using the portal for genotype-tissue expression project (GTEx Portal) and expression quantitative trait locus (eQTL) browser demonstrated that rs504348 (p = 4.2 × 10^−9^ and 2.1 × 10^−4^, respectively) modulates the expression of *ABCC1* in whole blood, which supports the *cis*-eQTL effect of rs504348 (Table 5).

## 3. Discussion

In this study, we investigated the *ABCC1* promoter SNP rs504348 by measuring plasma-induced *ABCC1* mRNA expression levels, and rs504348 *ABCC1* promoter methylation in CF subjects with differing *CFTR* genotypes. We correlated these molecular measures with clinical status to better understand the molecular mechanisms linking increased expression of *ABCC1* and improved lung function observed in CF patients. Previous studies reported that the rs504348 polymorphism in the *ABCC1* promoter has been reported to impact lung function [22,23], *ABCC1* mRNA expression [37,38], and CF disease severity [23]. We found higher plasma-induced *ABCC1* mRNA expression in CF subjects (Figure 1), but no significant association between rs504348 and clinical markers of CF disease severity (Table 3).

A prior study found that the rare CC genotype in CF is associated with a younger age at which FEV1 <60% predicted was first observed and a younger age of chronic infection with *P. aeruginosa* [23]. We analyzed the rs504348 genotypes and various markers of CF disease severity, such as ESCF classification [39,40], pancreatic status, and colonization with *P. aeruginosa* (Table 3), but found no significant association. Although we reported a similar rs504348 frequency distribution as the previous study, we also observed a significant deviation from HWE. We analyzed data from the 1000 Genome Project [41] but the results again showed a significant deviation from HWE in both the global population and Utah residents with Northern and Western European ancestry. One possible source for the observed disequilibrium could be the population stratification [42,43]. Our study cohort was not case-matched by genetic ancestry and although our study had a relatively small sample size, which may influence HWE estimates [44], even the larger sample sizes used for the 1000 Genome Project estimates showed significant deviations from HWE (Table 3).

Despite the limitation of small sample size in our study, we showed that CF subjects with *CFTR* genotypes associated with pancreatic insufficiency were more likely to be colonized with *P. aeruginosa* or the mucoid form of *P. aeruginosa* than those with genotypes associated with pancreatic sufficiency (Table 2). Pancreatic insufficiency or sufficiency is genetically determined [45,46], and the correlation between *CFTR* genotype and pancreatic phenotype is well established [47,48,49,50]. Pancreatic insufficient CF subjects harboring two severe mutations were more likely to have severe disease than pancreatic sufficient CF subjects harboring either two mild mutations or a combination of a severe and a mild mutation [45,46]. Although the association between *CFTR* genotypes and pancreatic disease is well characterized, the association between *CFTR* genotype and pulmonary phenotypes is less understood, perhaps due to variable fluctuations in lung function in CF patients [3,51].

FEV1 appears to be the most clinically useful measurement of lung function and the best available predictor of survival in patients with CF [4,51,52,53]. We adopted the ESCF classification to assign CF subjects into disease severity groups based on FEV1 percent predicted estimates and age [39,40], and found that the majority of CF subjects assigned to the Mild or Normal/mild severity group tested negative for mucoid *P. aeruginosa* and were pancreatic sufficient. Both pulmonary function tests and *P. aeruginosa* cultures were performed and recorded simultaneously during the same clinic visit. Chronic colonization of *P. aeruginosa* is associated with an accelerated decline of FEV1% percent predicted [54,55,56]. Our results may reflect current pulmonary status, but they may not predict future outcome. Similar to the results of previous studies [50], our results suggest that the severity classification of *CFTR* mutations that classically apply to pancreatic phenotypes may also apply to pulmonary phenotypes.

We observed higher expression of plasma-induced *ABCC1* mRNA in CF subjects with rs504348 (CC/CG) compared to those with the ancestral GG genotype (Figure 1D). Others [22] have reported reduced transcriptional activity associated with the G allele of this polymorphism in four cell lines. The *cis*-eQTL effect of rs504348 has been validated in whole blood in previous genome-wide eQTL projects [37,38]. We showed that the distribution of rs504348 genotypes (Table 3) and expression levels (Figure 1D) were not significantly different between CF disease severity groups. Since *ABCC1* expression analysis was measured at the mRNA level, our study may not describe the potential impact of the SNP on ABCC1 protein activity. Further study is warranted to assess the correlation between CF disease severity groups and ABCC1 protein expression or activity.

Our in silico analysis using HaploReg v4.1 reported a high cluster of *ABCC1* promoter activity across various tissues (Table 5). Histone marks associated with active promoters (H3K4me3 and H3K9ac) and active enhancers (H3K4me1 and H3K27ac) [57] were enriched in the region spanning rs504348 in PBMCs (Figure 4). *ABCC1* is ubiquitously expressed in several tissues [58], and no histone marks associated with inactive or methylated *ABCC1* promoter in any tissue were reported by HaploReg v4.1 (Table 5). It is well established that the methylation of promoters is inversely proportional to their transcriptional activity [27,59,60]. As we observed no difference in *ABCC1* promoter methylation status among CF subjects (Figure 3), it appears that DNA methylation may not be responsible for regulating *ABCC1* expression in peripheral blood of CF patients. Although in a previous cancer study [28], no significant difference in *ABCC1* promoter methylation was observed between normal and cancerous pancreatic tissues, whether DNA methylation regulates *ABCC1* expression in tissues other than peripheral blood in CF remains unclear and warrants further study. 

Plasma-induced transcriptional signatures are capable of characterizing CF disease severity [31]. We observed plasma-induced *ABCC1* expression in PBMCs to be significantly higher with the plasma of CF subjects compared to that of healthy controls (Figure 1A). In CF, long-term treatment with azithromycin results in improved lung function [61,62,63]. The macrolide is suggested to be efficacious in CF via upregulation of *ABCC1* [23,64]. Similarly, previous studies [15] suggested that the improved lung function observed in CF patients following treatment with antitumor drugs was due to upregulation of *ABCC1*. Since our study cohorts comprised of a clinically diverse group of CF patients undergoing standard CF care, we were unable to ascertain whether any medication was solely responsible for the differences in *ABCC1* expression. It is worth noting that ABCC1 plays a crucial role in the efflux of several drugs conjugated with glutathione and other anions [22,58,65]. Polymorphisms in the glutathione S-transferase (*GST*) family of genes have been associated with CF disease severity [66,67]. Early death and more severe disease are typical for *GSTM1*-deleted CF patients [68]. An enzyme belonging to the GST family of enzymes produces glutathione adducts and helps the human body by forming a detoxification system against electrophilic compounds and oxidative stress [69]. Both *CFTR* (*ABCC7*) and *MRP1* (*ABCC1*) are suggested to share this function of detoxifying natural or xenobiotic conjugates by exporting glutathione conjugates [15]. Hence, these genes are likely capable of complementing each other [15,23,64]. Further, since many drugs are good substrates for *ABCC1*, its overexpression results in multidrug resistance, especially in cancer [22,58]. CF patients are known to have a high prevalence of *P. aeruginosa* [55,70,71], which readily evolves resistance after long-term treatment with antibiotics [72]. Multidrug resistance isolates of *P. aeruginosa* [73,74], and other bacteria [75,76,77] are common in CF, making CF therapy challenging. Future studies to assess increased expression of *ABCC1* in CF patients, and its association with multidrug resistance to antibiotics, may shed light on the molecular underpinnings of recurrent *P. aeruginosa* infection in CF.

In summary, we demonstrated that plasma-induced transcriptional signatures are useful tools for investigating candidate genes associated with CF. The higher *ABCC1* mRNA levels induced by the plasma of CF patients may be related to the development of multidrug resistance to CF therapy; the increased activity of *ABCC1* may complement diminished *CFTR*. Although we confirmed the eQTL effect of rs504348 on plasma-induced *ABCC1* expression in PBMCs, there was no correlation between CF phenotypes and rs504348 genotypes. Histone modifications rather than DNA methylation may play a role in regulating *ABCC1* expression. Further study to identify regulatory networks and impact of therapies on *ABCC1* expression and role in *CFTR* activity may enhance our understanding of the clinical heterogeneity in CF.

## 4. Materials and Methods

### 4.1. Study Sample Characteristics

All CF subjects and healthy controls were recruited at the Children’s Hospital of Wisconsin (Milwaukee, WI, USA) and the Ann & Robert H. Lurie Children’s Hospital of Chicago (Chicago, IL, USA) with approval from the relevant Institutional Review Boards (IRB# CHW 07/72, CTSI 847 and 2015-400 respectively). Peripheral blood was drawn into citrate dextrose solution A or K^+^ ethylenediaminetetraacetic acid (EDTA) anticoagulant for each CF subject and plasma was isolated using Ficoll Histopaque (Sigma-Aldrich Corporation, St. Louis, MO, USA). Genomic DNA was extracted using the Puregene DNA Isolation kit (Gentra Systems Inc., Minneapolis, MN, USA) according to the manufacturer’s recommendations.

**Figure 5:**
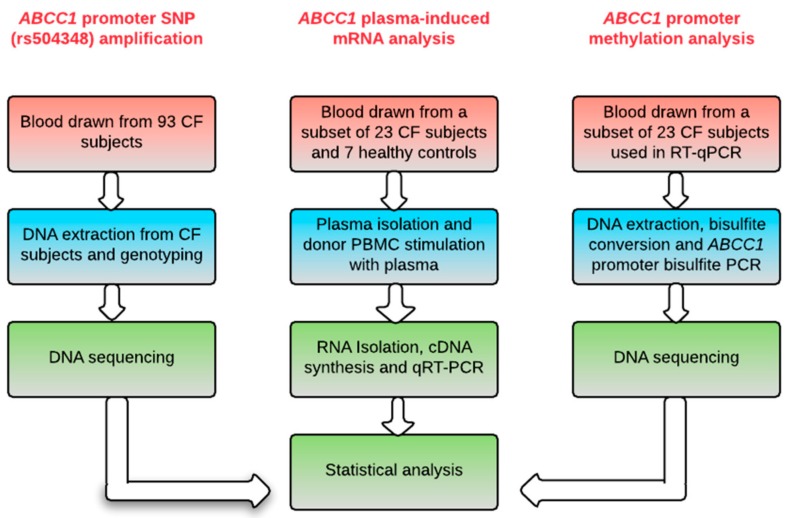
Schematic of experiment workflow. Peripheral blood collected from each CF subject was the primary source of genomic DNA and plasma. Genomic DNA extracted from 93 CF subjects was used for *ABCC1* promoter SNP amplification. Commercial donor PBMCs from Cellular Technology Limited (Shaker Heights, OH, USA) (UPN 727) stimulated with plasma from a subset of 23 CF subjects and an additional 7 healthy control samples were used for RNA isolation and gene expression analysis via qRT-PCR. *ABCC1* promoter methylation status of the subset of 23 CF samples used in qRT-PCR was determined using bisulfite PCR.

CF subjects were diagnosed based on the results of the pilocarpine iontophoresis sweat test (sweat chloride ≥ 60 mmol/L) and/or *CFTR* genotype, as previously described [31]. Demographic and clinical information, including pancreatic status, sweat chloride level, and *P. aeruginosa* infection status, was recorded for each CF subject. We adopted two definitions of CF disease severity categories in this study. First, the Epidemiologic Study of Cystic Fibrosis (ESCF) classification [39,40] was adopted to assign CF subjects into four severity groups based on measurements of FEV1 and age at recruitment. The four age groups/categories were: 6–12 years (severe, FEV1 ≤ 88.7% predicted; moderate, FEV1 > 88.7–94.5% predicted; mild, FEV1 > 94.5–99.0% predicted; very mild/normal, FEV1 > 99.0% predicted); 13–17 years (severe, FEV1 ≤ 76.5% predicted; moderate, FEV1 > 76.5–81.1% predicted; mild, FEV1 > 81.1–87.7% predicted; very mild/normal, FEV1 > 87.7% predicted); 18–29 years (severe, FEV1 ≤ 58.1% predicted; moderate, FEV1 > 58.1–63.9% predicted; mild, FEV1 > 63.9–70.7% predicted; very mild/normal, FEV1 > 70.7% predicted); and >30 years (severe, FEV1 ≤ 45.5% predicted; moderate, FEV1 > 45.5–50.9% predicted; mild, FEV1 > 50.9–59.8% predicted; very mild/normal, FEV1 > 59.8% predicted). The second definition of disease severity was based on the CF subject’s combination of *CFTR* class of mutations: the pancreatic insufficient group (severe disease) with CF subjects carrying functional mutations (class I, II, and III) [47] and a pancreatic sufficient (PS) group [47] (milder disease) with CF subjects who have a mild mutation (Class IV and V) [48,49]. We then assessed the association between disease severity groups and clinical status to determine whether our cohort data supported previous findings. A total of 93 CF subjects were genotyped at the *ABCC1* SNP rs504348 locus. Plasma-induced mRNA expression levels were analyzed from a subset of the CF subjects and healthy controls, and bisulfite PCR was used to assess promoter methylation status (Figure 5).

### 4.2. Genotyping

Genomic DNA extracted from 93 CF subjects were genotyped for *ABCC1* promoter SNP rs504348. A previously described set of forward (ABCC1F: 5′-CAGGATGAAATGAGGGCACAG-3′) and reverse (ABCC1R: 5′-GAAGCGCCTGGGATCTTTGG-3′) primers [22] were used for PCR with the GeneAmp^®^ PCR System 9700 (Applied Biosystems, Foster City, CA, USA). The 24 µL reaction mixture consisted of HotStarTaq Master Mix (Qiagen, Hilden, Germany), 5–15 ng/µL DNA, and 10 µM forward and reverse primers. Previously described [78] thermocycling conditions for the reaction were set: 1 min at 95 °C, followed by 35 cycles of 15 s at 95 °C, 15 s at 56 °C, 1.5 min at 72 °C, and a final cycle of 5 min at 72 °C. 

### 4.3. PBMC Culture, Total RNA Isolation, and qRT-PCRRT-qPCR

Commercial cryopreserved PBMCs from a healthy Caucasian HLA-A2 male donor (UPN727) were acquired from the ePBMC donor library provided by Cellular Technology Limited (CTL; Shaker Heights, OH, USA). PBMCs were washed and thawed according to the manufacturer’s recommendations. PBMCs were cultured for 9 h at 37 °C in 5% CO_2_ with 20% autologous plasma, CF plasma, CF parent plasma, or healthy control plasma using protocols previously described [31]. Following culture, total RNA was isolated using TRIzol Reagent (Invitrogen Life Technologies, Waltham, MA, USA) in accordance with the manufacturer’s recommendations prior to quantification on a ND-1000 spectrophotometer (Nanodrop, Wilmington, DE, USA). First-strand cDNA was synthesized from 5 ng of the isolated total RNA using the iScript™ cDNA Synthesis Kit (BioRad, Hercules, CA, USA) according to the manufacturer’s recommendations. qRT-PCR was conducted using the ABI 7500 Fast Real-time PCR system (Applied Biosystems) with Fast SYBR Green Master Mix (Applied Biosystems) using previously published primers for *ABCC1* mRNA [28]. The transcription of *GAPDH* was used as an internal control for normalization, and relative expression of the target gene for each sample was analyzed using the 2^−ΔΔ*C*t^ method [79].

### 4.4. Bisulfite PCR

EMBOSS Cpgplot (https://www.ebi.ac.uk/Tools/seqstats/emboss_cpgplot/) was used to predict CpG islands within the *ABCC1* promoter, which became the primary targets for methylation analysis after bisulfite PCR amplification. Bisulfite primers targeting CpG islands in the promoter region were designed using MethPrimer [80]. Genomic DNA extracted from the subset of 23 CF subjects used for qRT-PCR were used for methylation analysis. Genomic DNA was first converted to bisulfite-modified DNA using the EZ DNA Methylation-Gold™ Kit (Zymo Research, CA, USA) according to the manufacturer’s recommendations. Double-round bisulfite PCR was performed using the BF2 forward primer (5′-GTGATTTTGGGTAGAGGGAATTATT-3′) and the BR2 reverse primer (5′-CCCAAATCCTCCAAAACTTAAA-3′) in a 24 µL reaction mixture consisting of HotStarTaq Master Mix (Qiagen), 2 µL bisulfite-modified DNA, and 10 µM BF2 and BR2 primers. The second-round of PCR was performed using 1 µL of PCR products from the first round. The following thermocycling conditions were used: 10 min at 94 °C, followed by 35 cycles of 30 s at 95 °C, 30 s at 60 °C, 30 s at 72 °C, and a final cycle of 7 min at 72 °C.

### 4.5. SNP Annotation Data Query

We performed in silico analysis to assess the interaction between rs504348 with regulatory features and expression to validate our findings of our study. Functional annotation was performed using the 15-state and 25-state chromHMM models in HaploReg v4.1 [36]. HaploReg v4.1 (http://archive.broadinstitute.org/mammals/haploreg/) uses SNP information from the 1000 Genomes Project to map known genetic variants to data derived from ENCODE and Roadmap Epigenomics, unveiling SNP effects on regulatory features. Histone marks reported to contribute to the chromatin-state assignment at rs504348 by HaploReg v4.1 were visualized using the WashU Epigenome Browser v42 [81]. The *cis*-eQTL effect of rs504348 was explored using the GTEx Portal (http://gtexportal.org/home/) and the Blood eQTL browser [37].

### 4.6. DNA Sequencing and Statistical Analysis

Purified PCR amplicons were sequenced using ABI 3730 (Applied Biosystems, CA, USA) by ACGT Inc. (Wheeling, IL, USA). Overall, statistical analysis was conducted using SAS 9.4 (SAS Institute, Cary, NC, USA). Deviation from Hardy–Weinberg equilibrium was tested with the χ^2^ test for rs504348. A Shapiro-Wilk test was used to examine the normality of the continuous variables. Median and IQR were used if the data were not normally distributed. As appropriate, a *t*-test with equal or unequal variance was used for comparison between two groups. A Chi-square test or Fisher’s exact test was used to investigate the association between the categorical variables. *p* < 0.05 was considered significant. Bisulfite DNA sequences were analyzed with MethTools 2.0 [35].

## Figures and Tables

**Table 1:** Demographics of study subjects.

Parameter	CF Subjects
Samples (*n*)	93
Age in years, median (IQR)	10 (6,19)
Gender: Male, *n* (%)	44 (47.3%)
Pancreatic insufficient, *n* (%)	69 (74.2%)
*P. aeruginosa*, *n* (%)	54 (58.1%)
Mucoid *P. aeruginosa*, *n* (%)	32 (34.4%)
Sweat chloride (mmol/L), median (IQR)	103 (87,114)
FEV1 (%) predicted, median (IQR)	97 (72,111)
**ESCF disease severity classification**	*n* (%)
Normal/very mild	44 (47.3%)
Mild	10 (10.8%)
Moderate	7 (7.5%)
Severe	19 (20.4%)
Uncharacterized ^1^	13 (14.0%)
***CFTR* genotypes**	*n* (%)
F508del homozygotes	45 (48.4%)
F508del heterozygotes	40 (43.0%)
Other ^2^	8 (8.6%)

FEV1: Forced expiratory volume in 1 second; ESCF: Epidemiologic Study of Cystic Fibrosis. ^1^ Uncharacterized group consists of CF subjects less than 6 years old or no FEV1 (%) predicted values. ^2^ Other combinations of CFTR genotypes include: 394delTT/3272-26A>G, 711+5G>A/c.438_440delTCA, G551D/ R1070W, G542X/W1282X, G551D/R117H-7T, N1303K/R117H-7T, R117H-7T/R117H-7T and one unknown. *CFTR* mutations were defined according to the CFTR2 database or the Human Genome Variation Society (conventional name) nomenclature (www.hgvs.org) where available.

**Table 2:** Relationship of ESCF disease severity and clinical outcomes.

Parameter	Status	ESCF Disease Severity Classification *n* (%)				Pancreatic Status *n* (%)	
		Severe	Moderate	Mild	Normal/Very Mild	PI	PS
*P. aeruginosa status* *^,§^	Positive	15 (79)	7 (100)	5 (50)	23 (52)	45 (65)	9 (38)
Mucoid *P. aeruginosa* status *^,§^	Positive	12 (63)	5 (71)	3 (30)	12 (27)	29 (42)	3 (13)
Pancreatic status *	Pancreatic insufficient	19 (100)	6 (86)	10 (100)	28 (64)	--	--

ESCF: Epidemiologic Study of Cystic Fibrosis; PI: Pancreatic insufficient; PS: Pancreatic sufficient; --: not applicable. * *p* < 0.01, Moderate or Severe versus Mild or Normal/very mild; ^§^ *p* < 0.05, PI versus PS.

**Table 3:** Frequency distribution of rs504348

Parameter	Genotype			HWE *p*-Value	*p*-Value^3^
	CC	CG	GG		
**Populations ^1^**	***n* (%)**				
Global population	360 (14.4%)	658 (26.3%)	1486 (59.3%)	<0.0001	--
CEU	6 (6.1%)	22 (22.2%)	71 (71.7%)	<0.03	--
Study cohort	8 (8.6%)	24 (25.8%)	61 (65.6%)	<0.03	--
**ESCF Disease Severity Classification**	***n* (%)**				>0.17
Normal/very mild	5 (5.4%)	9 (9.7%)	30 (32.3%)	--	
Mild	1 (1.1%)	3 (3.2%)	6 (6.5%)	--	
Moderate	0 (0%)	1 (1.1%)	6 (6.5%)	--	
Severe	2 (2.2%)	3 (3.2%)	14 (15.1%)	--	
Uncharacterized 2	0 (0%)	8 (8.6%)	5 (5.4%)	--	
**Pancreatic Status**	***n* (%)**				>0.14
Pancreatic insufficient	5 (5.4%)	15 (16.1%)	49 (52.7%)	--	
Pancreatic sufficient	3 (3.2%)	9 (9.7%)	12 (12.9%)	--	
***P. aeruginosa***	***n* (%)**				>0.89
Positive	4 (4.3%)	14 (15.1%)	36 (38.7%)	--	
Negative	4 (4.3%)	10 (10.8%)	25 (26.9%)	--	
**Mucoid *P. aeruginosa***	***n* (%)**				>0.31
Positive	4 (4.3%)	10 (10.8%)	18 (19.4%)	--	
Negative	4 (4.3%)	14 (15.1%)	43 (46.2%)	--	

HWE: Hardy–Weinberg equilibrium; ESCF: Epidemiologic Study of Cystic Fibrosis; --: not applicable. ^1^ Global population and CEU (Utah residents with Northern and Western European ancestry) adopted from 1000 Genomes Project (http://www.ensembl.org). ^2^ The uncharacterized group consisted of CF patients less than 6 years old or have no FEV1 (%) predicted values. ^3^ *p*-value estimated using Fisher’s Exact tests.

**Table 4:** Clinical and demographic information for subjects included in expression analysis.

Parameter	CF Subjects	Healthy Controls ^1^
Samples, *n*	23	7
Age in years, median (IQR)	9.4 (6.7, 17.3)	26.9 (7.0, 27.1)
Pancreatic insufficient, *n* (%)	13 (56.5%)	--
***ABCC1* genotypes**	***n* (%)**	
Ancestral GG	5 (21.7%)	--
rs504348 (CC/CG)	18 (78.3%)	--
***CFTR* genotypes ^2^**	***n* (%)**	
F508del homozygotes	11 (48.0%)	--
F508del heterozygotes	11 (48.0%)	--
G542X/W1282X	1 (4.0%)	--

^1^ All healthy control volunteers were unrelated and included for comparison with CF subjects. ^2^ *CFTR* mutations were defined according to the CFTR2 database or the Human Genome Variation Society (conventional name) nomenclature (www.hgvs.org) where available; --: not applicable.

**Table 5:** In silico predictions for rs504348 and variant (rs762775) in high linkage disequilibrium (LD).

hg38 Chromosome (Position)	SNP	SNPs High LD (*r*^2^)	Promoter Histone Marks	Enhancer Histone Marks	DNAse	Proteins Bound	Motifs Changed	eQTL Hits
16 (15949317)	rs504348	1	24 tissues, including PBMCs	Many tissues including PBMCs	30 tissues	8 bound proteins	4 altered motifs	2 hits ^2^
16 (15954300)	rs762775 ^1^	0.62		13 tissues	1 tissue		2 altered motifs	2 hits

^1^ rs762775 was the only SNP detected to be in high (*r*^2^ > 0.6) linkage disequilibrium (LD) with rs504348. ^2^ The 2 eQTL hits are results from GTEx Portal and Blood eQTL. Both results show rs504348 significantly (*p* < 0.05) impacts *ABCC1* expression.
